# Behavioral variant of frontotemporal dementia or frontal variant of Alzheimer's disease? A case study

**DOI:** 10.1590/1980-57642018dn13-030015

**Published:** 2019

**Authors:** Leonardo Cruz de Souza, Luciano Inácio Mariano, Renata Freire de Moraes, Paulo Caramelli

**Affiliations:** 1 Universidade Federal de Minas Gerais Programa de Pós-Graduação em Neurociências Belo HorizonteMG Brazil Programa de Pós-Graduação em Neurociências, Universidade Federal de Minas Gerais (UFMG), Belo Horizonte, MG, Brazil.; 2 UFMG Faculdade de Medicina Departamento de Clínica Médica Belo HorizonteMG Brazil Departamento de Clínica Médica, Faculdade de Medicina, UFMG, Belo Horizonte, MG, Brazil.; 3 Hermes Pardini Belo HorizonteMG Brazil Hermes Pardini, Belo Horizonte, MG, Brazil.

**Keywords:** Alzheimer's disease, frontotemporal dementia, atypical Alzheimer's disease, biomarkers, doença de Alzheimer, demência frontotemporal, doença de Alzheimer atípica, biomarcadores

## Abstract

Alzheimer's disease (AD) has heterogeneous clinical presentations. Amnestic progressive disorder leading to dementia is the most typical, but non-amnestic presentations are also recognized. Here we report a case of frontal variant of AD. A right-handed woman, aged 68 years, was referred for progressive behavioral disorders and personality changes. She had a corroborated history of dietary changes, hyperorality, impulsivity, affective indifference and apathy, with functional impairment. Cognitive assessment yielded severe executive deficits. Positron emission tomography with fluorodeoxyglucose showed marked hypometabolism in frontotemporal regions, with relative preservation of parietal regions. CSF AD biomarkers showed low Aβ_42_, high Tau and high P-Tau. The patient fulfilled criteria for probable behavioral variant frontotemporal dementia. However, considering the AD pathophysiological signature on CSF biomarkers, a diagnosis of frontal variant of AD was established. In the perspective of disease-modifying therapies, it is important to identify atypical Alzheimer presentations, as these patients may be candidates for specific treatments.

## RESUMO

A doença de Alzheimer (DA) tem apresentações clínicas heterogêneas. Amnésia progressiva associada a demência é a forma mais comum, mas apresentações não-amnésticas são também reconhecidas. Relatamos um caso de variante frontal da DA. Um mulher destra, de 68 anos, consultou-se devido a transtornos comportamentais progressivos, com alterações de personalidade. Modificações de padrão alimentar, hiperoralidade, impulsividade, indiferença afetiva e apatia, com declínio funcional, foram corroboradas pela família. A avaliação cognitiva evidenciou grave disfunção executiva. A tomografia de emissão de pósitrons com flúordeoxiglicose revelou proeminente hipometabolismo em regiões frontotemporais, com relativa preservação de regiões parietais. A análise de biomarcadores de DA no líquido cefalorraquidiano mostrou redução de níveis de Aβ_42_, com aumento de níveis de Tau e P-Tau. A paciente preencheu critérios diagnósticos para demência frontotemporal (variante comportamental) provável. Contudo, considerando o perfil de biomarcadores em favor de fisiopatologia da DA, o diagnóstico de variante frontal de DA foi estabelecido. Na perspectiva de tratamentos modificadores da doença, é crucial identificar apresentações atípicas da DA, visto que esses pacientes podem ser candidatos a terapias específicas.

Frontotemporal dementia (FTD) is the second most common cause of early-onset dementia, presenting three main phenotypes: behavioral variant (bvFTD), non-fluent progressive aphasia and semantic dementia.^1^ bvFTD is the most frequent phenotype and is characterized by progressive behavioral and personality disorders, featuring disinhibition, apathy, loss of empathy, dietary changes, and ritualistic/stereotypical behaviors.^2^ bvFTD may also present cognitive deficits, with impairment in executive functions and social cognition, yet relative sparing of episodic memory and visuoconstructive abilities.^2^ From a pathological point of view, bvFTD is classified as frontotemporal lobe degeneration (FTLD). FTLD with tauopathy or FTLD with *TAR DNA-binding protein of 43 kDA* (*TDP-43*) inclusions are the most common underlying findings among bvFTD patients.^3^ Besides FTLD pathology, bvFTD may be due to Alzheimer's pathology.^3^^-^5 Indeed, it has been recognized that the frontal^6^ or behavioural/dysexecutive^5^ variant represents an atypical presentation of Alzheimer's disease (AD). Atypical phenotypes of AD may be identified *in vivo* by pathophysiological markers, such as CSF biomarkers or amyloid imaging.^6^ Here, we present a case of frontal variant of AD, with CSF Alzheimer signature.

## CASE REPORT

A right-handed woman, aged 68 years, a retired bank agent with 16 years of education, was referred for neurological evaluation in 2016, for progressive behavioral disorders and personality changes evolving for approximately six months. Her family reported dietary changes, with marked preference for sweet foods. Hyperorality was also evident, with increased consumption of cigarettes. She also exhibited oral exploration, picking up inedible objects from the floor and putting them in her mouth. There was ritualistic behavior, with a tendency to repetitively clean and organize things in her house. The family reported that she became strict with time schedules. She had excessive expenses with perfumes and body creams, using them compulsively. Mild affective indifference was observed. She also manifested poor judgment abilities, with marked concrete thinking. The family reported no memory deficits or spatial disorientation, and autonomy for activities of daily living was globally preserved.

She had a depressive episode at the age of 41, without recurrence. She had been followed by a psychiatrist over the last two years, due to anxious disorder. There was no history of hallucinations, delusions, seizures, head trauma, or alcohol abuse. She had no family history of dementia or motor neuron disease.

Neurological examination was normal, without parkinsonism or eye movement abnormalities. Myoclonus, fasciculations and muscle atrophy were absent. There were no frontal release signs.

The patient underwent two formal cognitive assessments (2016 and 2018; Table 1). Initial evaluation (2016) showed no impairment of global cognitive efficiency (Mini-Mental State Exam [MMSE]=29/30),^7^ with preserved time/space orientation. Performance on the Rey Auditory Verbal Learning Test (RAVLT)^8^ demonstrated preservation of encoding, learning, retrieval and recognition. Her performance on a visual episodic memory test^9^ was also normal. Planning deficits were detected on the copy of Rey complex figure and on the Tower of London test. Verbal fluencies were preserved, both in phonemic and categorical modalities. Reading and writing abilities were spared, with preserved comprehension of written language.

**Table 1:** Cognitive data.

	2016	2018
Mini-Mental State Exam ( /30)	29	26
• Time Orientation ( /5)	5	4
• Space Orientation ( /5)	5	5
• Immediate Memory ( /3)	3	3
• Calculation ( /5)	4	4
• Recall ( /3)	3	1
• Language ( /8)	8	8
• Drawing ( /1)	1	1
Rey Auditory Verbal Learning Test (RAVLT) - A1 ( /15)	5	3
Rey Auditory Verbal Learning Test (RAVLT) - A2 ( /15)	7	7
Rey Auditory Verbal Learning Test (RAVLT) - A3 ( /15)	10	7
Rey Auditory Verbal Learning Test (RAVLT) - A4 ( /15)	12	9
Rey Auditory Verbal Learning Test (RAVLT) - A5 ( /15)	12	10
Rey Auditory Verbal Learning Test (RAVLT) - B1 ( /15)	3	2
Rey Auditory Verbal Learning Test (RAVLT) - A6 ( /15)	11	9
Rey Auditory Verbal Learning Test (RAVLT) - A7 ( /15)	13	7
Rey Auditory Verbal Learning Test (RAVLT) - Recognition ( /15)	13	4
Rey Auditory Verbal Learning Test (RAVLT) - Sum A1-A7	46	36
Figures Memory Test (Incidental) ( /10)	6	6
Figures Memory Test (Immediate) ( /10)	10	8
Figures Memory Test (Learning) ( /10)	10	8
Figures Memory Test (5-minute recall) ( /10)	10	7
Figures Memory Test (Recognition) ( /10)	10	10
Rey-Osterrieth Complex Figure Test (Copy)	25	35
Rey-Osterrieth Complex Figure Test (Recall)	14	4
Tower of London	26	26
Letter Fluencies (F, A, S)	35	37
Categorical fluency (Animals)	14	10
Categorical fluency (Fruits)	14	17

In 2018, the patient had subnormal performance on the MMSE (26/30). The performance on the Mattis scale^10^ was normal (142/144), but global cognitive efficiency was impaired (79/100) on the Addenbrooke Cognitive Examination - Revised Version (ACE-R),^11^ due to significant deficits in verbal fluency and figure naming, with relative preservation on memory tests. Scores on the RAVLT were markedly worse than in 2016. Similarly, scores on the visual episodic memory test^9^ were also worse than previously. Performances on verbal fluencies remained globally stable. Flexibility and conceptualization were impaired on the modified Wisconsin Card Sorting Test.^12^ The patient presented attentional and working memory impairments, and also deficits of inhibitory control on the Hayling test. In summary, she exhibited a full dysexecutive syndrome.

This second cognitive assessment also included tests of social cognition (Table 2). The patient had normal performance on two tests of theory of mind, the *faux-pas* test from the Social and Emotional Assessment^13^ and the Theory of Mind - 15 (TOM-15), a false-belief test.^14^ The total score on the emotion recognition test^15^ was normal (28/35), but she had marked deficit in the recognition of fear (0 out 5).

**Table 2:** Social Cognition Assessment (August 2018).

Test	Raw score
Faux-Pas Test: Total Score ( /40)	38
• Stories with Faux-Pas ( /30)	28
• Stories without Faux-Pas ( /10)	10
• Faux-Pas Test - Comprehension ( /10)	10
False-Belief Test (Theory of mind - 15): total score ( /15)	12
• False-Belief Test (Theory of mind - 15): first order ( /8)	7
• False-Belief Test (Theory of mind - 15): second order ( /7)	5
• False-Belief Test (Theory of mind - 15): comprehension ( /15)	15
Facial Emotion Recognition Test - Total score ( /35)	28
• Facial Emotion Recognition Test - Happiness ( / 5)	5
• Facial Emotion Recognition Test - Surprise ( / 5)	5
• Facial Emotion Recognition Test - Disgust ( / 5)	5
• Facial Emotion Recognition Test - Fear ( / 5)	0
• Facial Emotion Recognition Test - Anger ( / 5)	5
• Facial Emotion Recognition Test - Sadness ( / 5)	4
• Facial Emotion Recognition Test - Neutral ( / 5)	4

The patient underwent a complete blood exam in order to exclude causes of non-neurodegenerative dementias, without abnormalities.

**Figure 1:**
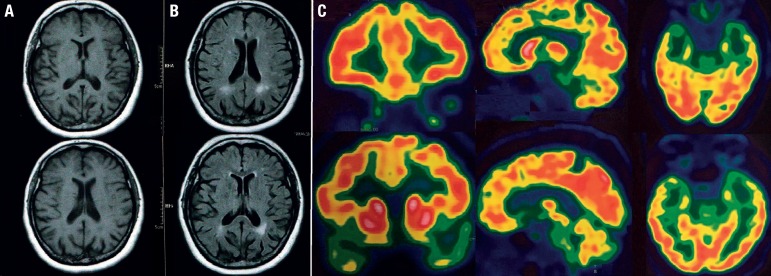
A. Magnetic resonance imaging (MRI) of brain showing moderate parietal atrophy and no focal frontotemporal atrophy. B. Moderate white matter disease on brain MRI. C. Positron emission tomography with fluorodeoxyglucose (PET-FDG) showing marked hypometabolism in temporal poles and anterior cingulate; there is mild hypometabolism in the orbitofrontal cortex, medial temporal regions (including hippocampus) and temporo-parietal cortex. No hypometabolism is evident in the precuneus and posterior cingulate.

Brain MRI (January/2017) revealed mild parietal atrophy, without medial temporal or frontotemporal atrophy. The patient underwent another brain MRI thirteen months later (February/2018), which disclosed a similar pattern, but with more pronounced parietal atrophy (Figure 1A). There were moderate periventricular white matter lesions (Fazekas 2 - Figure 1B). The burden and topography of these lesions were considered very unlikely to be the cause of the full behavioral syndrome, especially impulsivity, hyperorality, ritualistic behavior and dietary changes.

Positron emission tomography with fluorodeoxyglucose ([PET-FDG] - November/2018) showed marked hypometabolism in temporal poles and the anterior cingulate (Figure 1B). There was mild hypometabolism in orbitofrontal cortex, medial temporal regions (including hippocampus) and temporo-parietal cortex. Importantly, there was no hypometabolism in the precuneus and posterior cingulate.

Lumbar puncture was performed for clinical purposes and routine analysis was unremarkable. CSF AD biomarkers were measured and showed low Aβ_42_ (289 pg/mL; cut-off >600 pg/mL), high Tau (473 pg/mL; cut-off <350 pg/mL) and high P-Tau (74 pg/mL; cut-off <60 pg/mL), in favor of an AD pathophysiological process.

During a 24-month clinical follow-up, there was deterioration in cognitive abilities and mild loss of autonomy (Pfeffer Functional Activities Questionnaire=9/30). She had taken out a large financial loan, and the family had to take control of her finances. The patient clinically developed severe apathy (Starkstein Apathy Scale=32/42) and reduced personal hygiene. The patient has been treated with antipsychotics for behavioral control. Treatment with cholinesterase inhibitors was introduced, without clinical improvement.

The patient fulfills criteria for probable bvFTD according to consensus diagnostic criteria:^2^ 1) a corroborated history of initial progressive behavioral and personality changes, with dietary changes, hyperorality, impulsivity, ritualistic behavior, affective indifference and apathy; (2) presence of severe executive deficits; 3) marked hypometabolism on PET-FDG in frontotemporal regions (temporal poles, anterior cingulate), with relative preservation of parietal regions; 4) Functional impairment. Considering the AD pathophysiological signature on CSF biomarkers, a diagnosis of frontal variant of AD was established.

## DISCUSSION

AD has heterogeneous clinical presentations. Amnestic progressive disorder leading to dementia is the most typical,^16^ but non-amnestic presentations, such as posterior cortical atrophy, logopenic aphasia and frontal variant, are also recognized.^6^ Here, we report a patient fulfilling consensus criteria for probable bvFTD, but presenting CSF biomarker evidence of underlying Alzheimer's pathophysiology. According to the recently proposed biological definition of AD,^17^ the patient can be classified as A+ (abnormal CSF Aβ_42_), T+ (abnormal CSF P-Tau) and (N)+ (hypometabolism on PET-FDG and abnormal CSF Tau), thus meeting criteria for Alzheimer's continuum.^17^

The frequency of frontal variant of AD is unknown. Most clinicopathological studies have found that bvFTD is rarely associated to Alzheimer pathology.^3^^,^^18^ However, it should be pointed out that comorbid Alzheimer-related pathological changes may be observed in bvFTD patients with confirmed FTLD.^19^^,^^20^ Therefore, it is possible that the Alzheimer CSF biological signature in our patient is due to comorbid association between AD and FTLD.

Patients with Alzheimer variants are usually younger than patients with typical AD. In the present case, behavioral symptoms started after the age of 65 years; clinicians should be aware of atypical Alzheimer presentations even in older subjects.

Patients with atypical Alzheimer presentations do not exhibit typical distribution of neurofibrillary pathology, initially involving transentorhinal cortex, hippocampus and then spreading through association cortices.^16^ On the contrary, it has been demonstrated that patients with frontal variant of AD have greater pathological findings in frontal regions than typical AD.^21^^-^23 Molecular neuroimaging studies with tau markers reinforce that there are distinct topographical patterns of neurodegeneration across different presentations of AD.^24^

There is an overlap of cognitive deficits between bvFTD due to FTLD and frontal variant of AD. Although data exists suggesting that patients with behavioral AD have worse performance on episodic memory tests than bvFTD-FTLD patients,^5^ frank amnesia may also be observed in bvFTD.^25^ Our patient had normal scores on both visual and verbal episodic memory tests on the first cognitive assessment, but her performance declined over time.

Deficits in facial emotion recognition, theory of mind and knowledge of social norms have been reported in patients with frontal variant of AD,^26^^,^^27^ rendering it difficult to differentiate from bvFTD-FTLD. In the present case, the patient had isolated deficit in fear recognition. There was no deficit on theory of mind tasks. More studies are warranted to investigate social cognition in patients with frontal AD, and to assess the diagnostic accuracy of social cognition tests in the differential diagnosis between frontal AD and bvFTD.

Neuroimaging is a crucial tool in the investigation of cognitive-behavioral disorders, as specific atrophic patterns are associated with distinct clinical diagnoses. Compared to bvFTD-FTLD, patients with behavioral/dysexecutive variant of AD have greater parietal atrophy.^5^ Our patient had posterior involvement on brain MRI, thus supporting the diagnosis of atypical AD. However, she did not exhibit hypometabolism in the precuneus or posterior cingulate, which has been found in behavioral/dysexecutive variant.^5^ Conversely, she had marked hypometabolism in temporal poles and anterior cingulate, with mild involvement of the orbitofrontal cortex. These findings are commonly observed in bvFTD and are closely related to the patient's behavioral symptoms (apathy, impulsivity, dietary changes). We consider that functional neuroimaging is a helpful tool to elucidate behavioral symptoms in patients with normal or borderline findings on structural imaging.

In summary, the present report illustrates the phenotypical heterogeneity of AD. Atypical Alzheimer variants represent a clinical diagnostic challenge. In the perspective of treatments targeting amyloidosis and Alzheimer-related tauopathy, it is important to identify atypical Alzheimer presentations such as frontal variant, as these patients may be candidates for specific disease-modifying treatments.
